# Chapter 12: Decompressive Craniectomy: Long Term Outcome and Ethical Considerations

**DOI:** 10.3389/fneur.2019.00876

**Published:** 2019-09-06

**Authors:** Kevin Kwan, Julia Schneider, Jamie S. Ullman

**Affiliations:** Department of Neurosurgery, Donald and Barbara Zucker School of Medicine at Hofstra/Northwell, Hempstead, NY, United States

**Keywords:** outcomes—health care, decompressive craniecotmy, intracranial hypertension, medical ethics, traumatic brain injury (craniocerebral trauma)

## Abstract

Decompressive craniectomy (DC) for the treatment of severe traumatic brain injury (TBI) has been established to decrease mortality. Despite the conclusion of the two largest randomized clinical trials associating the effectiveness of decompressive craniectomy vs. medical management for patients with traumatic brain injury (TBI), there is still clinical equipoise concerning the usefulness of DC in the management of refractory intracranial hypertension. Primary outcome data from these studies reveal either potential harm or that decreased mortality only leads to an upsurge in survivors with severe neurologic incapacity. In this chapter, we seek to review the results of the most recent clinical trials, highlight the prevailing controversies, and offer potential solutions to address this dilemma.

## Background

Averting cerebral hypoxia and hypotension as well as subsequent secondary injury are the key aims of management following severe traumatic brain injury (TBI). Cerebral ischemia can occur through reduced autoregulation after neural insults leading to disturbance of the usual homeostatic mechanisms (1). This can result in a malicious sequence of amplified intracranial hypertension, reduced cerebral blood flow, and metabolic derangement (2, 3). As intracranial pressure continues to increase, subsequent cerebral herniation can result in poor patient outcomes (4).

Decompressive craniectomy (DC), the surgical removal of a portion of the skull, has been used for many years in patients with TBI (5). In patients with raised intracranial pressure (ICP), DC has been described to increase cerebral perfusion and oxygenation leading to enhanced clinical outcome in patients with intractable hypertension (6, 7).

The controversy in the role of DC in severe TBI stems from the contradictory results of the latest randomized controlled trials (RCTs) (8–12). Some proponents against DC for TBI suggest it may simply increase the subset of subjects who survive but remain neurologically non-functional with subsequent poor quality-of-life (11, 13). Opinion varies concerning the operating techniques used in patients undergoing DC. Over the past 30 years, several clinical investigations and observational studies have tried to address this through examining craniectomy size, craniectomy vs. craniotomy, and surgery time (14–18). Thus, despite the conclusion of the two largest RCTs comparing the efficacy of DC vs. medical management for patients with TBI, there is still clinical equipoise regarding the roll of DC in the management of refractory ICH (11, 12).

## Existing Randomized Clinical Trials—DECRA and RESCUEicp Trials

“The Early Decompressive Craniectomy in Patients With Severe Traumatic Brain Injury” (DECRA trial) RCT, associated bifronto-temporo-parietal DC to primary medical management for refractory ICH, with refractory ICP defined *as* >*20 mm Hg within an hour window for* >*15 min*. The investigation employed subjects in 15 tertiary care hospitals in three countries between 2002 and 2010 (12). The DC group included 73 patients and the medical management group included 82. The study found inferior scores with regard to Extended Glasgow Outcome Scale (GOS-E), for subjects if enrolled for DC, despite these patients having had lower ICP and fewer ICU days, than for those having received standard care at 6 months post-injury. Mortality at 6 months for DC was 19% vs. for medical management 18% for medical management (not statistically significant) and GOS-E showed a trend toward worse outcomes if enrolled for DC. Limited inclusion criteria, including the threshold for refractory ICP (ICP > 20 for 15 min within a 1-h period), raised inquiries regarding the generalizability of the results (12).

“The Randomized Evaluation of Surgery with Craniectomy for Uncontrollable Elevation of Intracranial Pressure” (RESCUEicp) sought to resolve these issues. The inclusion criteria were refined to include subjects with intracranial mass lesions of the traumatic subtype. In addition, the definition of refractory ICP was re-defined as >*25 mm Hg between 1 and 12 h in duration* (11). On subject presentation, radiographic imaging was reviewed and stratification for either single-sided or bilateral craniectomy based on the clinical judgment of the surgeon. Subjects enrolled in the medical treatment arm could receive further barbiturates as needed to dampen ICP. If continued clinical worsening occurred, subjects could also cross-over and receive a subsequent decompressive craniectomy. Six months GOS-E was utilized as the primary outcome. Twelve months GOS-E was the secondary outcome. This RCT remarkably revealed improved ICP and better mortality rates overall. There was a notable increase in the subset of patients with poor GOS-E, a score usually associated with poor quality of life.

While results from the RESCUEicp trial established an improvement in mortality for DC at 6 months, they also displayed increased rates of vegetative state and disability than medical care.

Of note, the investigation also completed a subsequent analysis looking at the percentage of subjects that had GOS-E scores between 4 and 8. This patient subset was deemed as “favorable” as they would be independent at home or better. At 6 months, there were no significant differences between the GOS-E scores between the DC and medically treated subjects (42.8 and 34.6%, respectively; *P* = 0.12), but when looking at the 12-months data, a significant trend toward benefit from DC begins to emerge (45.4 vs. 32.4%; *P* = 0.01) (11). A comparison of DECRA and RESCUEicp is found in Table 1.

**Table 1:** DECRA vs. RESCUEicp trial comparison.

	**DECRA**	**RESCUEicp**
Surgical group	73	202
Medical group	82	196
Age (years)	15–60	10–65
Number tertiary centers	15	52
Duration study (year)	2002–2010	2004–2014
Surgical proecedure	Bilateral	Bilateral or Unilateral
Criteria for DC	ICP > 20 mmHG, within 1h, for >15 min	ICP > 25 mmHg, between 1 and 12 h
Duraton follow up	6 months	24 months

## Ethical Considerations for Patients and Families: Survival with Unfavorable Outcome

Grounded on the current clinical data using primary outcome measures ranging only to 6 months, it would seem that utilization of DC for TBI can be a lifesaving intervention. The major concern however, is that this surgical choice may merely save lives at the expense of existence with severe disability and, thus, a poor quality of life (19, 20).

Survey studies have been initiated on patients who have experienced DC in the context of stroke, investigated their outcome satisfaction and whether surgery would have been acceptable initially. If their reply was generally affirmative, this answer would surmise retrospective consent (21). In one study, 28 patients were followed after undergoing DC to assess long-term outcomes. Retrospective consent to DC was achieved in roughly four out of five patients. Notably they mention that patients that achieved modified Rankin scores of four or better tended to provide retrospective consent (21). A conceivable explanation of these outcomes may be that these patients were able to adapt to and accept their neurologic disability. Indeed, quality of life perceptions are ultimately patient specific, with perceptions of whether life is perceived to be “worth living” is dependent on the individual context (22).

It is important to have patients discuss their life-support preferences with their health care delegates. Shared decision making should be emphasized regarding medical and surgical options, possible outcomes from involvement, and truthful quality of life goals following recovery. Patients should be aware that if they are not able to provide consent at the time of a severe TBI with no health care proxy available, then surgery may be performed at the discretion of the surgeon/ health care team.

## Future Considerations: Utilizations of Long-Term Outcome Data

The current clinical trial results strongly suggest the disadvantages of restricting follow up to <1 year. The lack of encouraging clinical evidence to back the use of DC in TBI may be, in part, due to the use of 6-months primary outcome data. This is especially relevant in the context of severe TBI, as patients often require extended time (12–24 months, or more) for functional recovery.

There is evidence of an advantage to using long term outcome metrics to evaluate the role of DC for TBI. Investigations, however are limited only to retrospective cohort studies, as RCTs to date have only published up to 12-months outcome data. Of the studies that address longer-term follow up, there have been notable improvements in outcome (23, 24). One study found in a cohort not included in the DECRA trial, that roughly half of patients (*N* = 176) had a one-point improvement in the GOS-E score between 6 and 18 months after DC. Of the 59 patients that had unfavorable outcome 6 months following surgery (defined as severe disability or worse), 25% (*n* = 15) improved to favorable outcome (defined as moderate disability or better) at 18-months follow up (23). Another investigation found an 11.6% significant increase in favorable outcome between three months and 2 years follow up (*n* = 60) (24).

In other neurosurgical literature, the utility of RCT's with long-term follow up has shed light in guiding the treatment paradigm for patients presenting with ruptured intracranial aneurysms. Notably, the 9-years outcome data for the International Subarachnoid Aneurysm Trial (ISAT) helped address the controversy of aneurysm stability treated with endovascular intervention, with long term follow-up results demonstrating that the risk of re-bleeding with this intervention was low (25).

TBI symptomatology may persevere for decades harming cognitive capabilities and psychosocial functioning, advocating for looking at quality of life (QoL) outcomes for a duration of more than the standard 3 years in order to obtain accurate clinical results (26). There is also a subset of patients whose outcomes may worsen over time due to structural impairments of the brain, progression of brain atrophy and microstructural changes (27). We recognize the barriers faced by the authors of the DECRA and RESCUEicp trials when conducting their respective large scale RCTs in regards to the restrictive fiscal barriers imposed by RCT subsidy (28). However, we propose that it is a necessity to include funding for 12–36 months follow-up to support research coordinators and data management. Furthermore, it may be of benefit to streamline outcome variables in order to mitigate patient attrition and to utilize web-based techniques to streamline follow up (29).

## Conclusions

In spite of the conclusion of the two largest RCTs equating the efficacy of DC compared to medical management for patients with TBI, the recommendations and indications for the use of DC in the context of refractory ICH remains highly debatable. The DECRA study displayed no advantage from early bifrontal surgical DC to reduce ICP in diffuse TBI, though the applicability of the results were questionable owing to restrictive inclusion criteria. The RESCUEicp trial brought a new perspective to these issues by including more frequently encountered patient conditions and by raising the threshold for refractory ICH (>25 mm Hg for 1–12 h). The RESCUEicp trial showed that DC in patients who failed initial treatment measures was associated with lower mortality than in patients who underwent medical management. However, at 6-months, a greater number of subjects in the DC arm than in the medical treatment arm, were in a vegetative state or reliant on others for activities of daily living.

While these results may underscore the belief that improvements in mortality from emergent lifesaving procedures do not necessarily correlate with enhancements in quality of life, there is concern for relying solely on 6-months primary outcome measures to define the effectiveness of a treatment for a condition (severe TBI) that demonstrates ongoing recovery for 12–24 months, or longer. Careful evaluation of the 12-months outcome for RESCUEicp suggests improvement in the DC cohort given that the upper severe disability group, by definition, had partial independence at home. Twenty-four months follow-up is anticipated to be published after the data is examined. However, the conclusions thus far argue for greater inspection in selecting the criteria of patients chosen for DC and for enhancement in diagnosis and treatment through further investigation and technological innovation.

It is worth noting that discussions regarding decompressive craniectomy should also include the optimal timing of cranioplasty (replacement of the bone flap or artificial substitute) to restore then normal anatomy of the cranium. Risks of prolonged trephanation may include focal neurological deficits, or stored bone flap erosion. Hydrocephalus and extra-axial hygromas can occur due to altered cerebrospinal fluid dynamics. Unfortunately an optimal time of cranioplasty has not yet been delineated but early cranioplasty has been shown to result in shorter operative times and decrease costs (30).

Due to the complicated discussions regarding patient outcomes and quality of life goals, it is unlikely that a single algorithm can be followed to guide patients and their families through the difficult sequela of TBI. Additionally, the ethical concerns may also vary based on the unique cultural beliefs, faiths and medical economics of the patient's geographic location. Regrettably, the acute clinical setting in which these matters need to be deliberated is inadequate and psychologically stressful. However, it is necessary to have early, comprehensive discussions with families regarding the risks and benefits of treatment. These conversations should take into account the potential prognosis for recovery and, whenever possible, include the patients' prior wishes and tolerance for disability.

## Author Contributions

KK and JS both performed research and wrote the text. JU, the senior author, conceived of article theme, guided structure, and edited manuscript.

### Conflict of Interest Statement

The authors declare that the research was conducted in the absence of any commercial or financial relationships that could be construed as a potential conflict of interest.

## References

[1] Kakar V Nagaria J John Kirkpatrick P. The current status of decompressive craniectomy. Br J Neurosurg. (2009) 23:147–57. 10.1080/02688690902756702 19306169

[2] Xi G Keep RF Hoff JT. Pathophysiology of brain edema formation. Neurosurg Clin North Am. (2002) 13:371–83. 10.1016/S1042-3680(02)00007-4 12486926

[3] Stocchetti N Maas AI. Traumatic intracranial hypertension. N Engl J Med. (2014) 371:972. 10.1056/NEJMc1407775 25184881

[4] Dunn LT. Raised intracranial pressure. J Neurol Neurosurg Psychiatry. (2002) 73 (Suppl. 1):i23–7. 10.1136/jnnp.73.suppl_1.i23 12185258 PMC1765599

[5] Guerra WK Gaab MR Dietz H Mueller JU Piek J Fritsch MJ. Surgical decompression for traumatic brain swelling: indications and results. J Neurosurg. (1999) 90:187–96. 10.3171/jns.1999.90.2.0187 9950487

[6] Bor-Seng-Shu E Figueiredo EG Amorim RL Teixeira MJ Valbuza JS de Oliveira MM. Decompressive craniectomy: a meta-analysis of influences on intracranial pressure and cerebral perfusion pressure in the treatment of traumatic brain injury. J Neurosurg. (2012) 117:589–96. 10.3171/2012.6.JNS101400 22794321

[7] Eberle BM Schnuriger B Inaba K Gruen JP Demetriades D Belzberg H. Decompressive craniectomy: surgical control of traumatic intracranial hypertension may improve outcome. Injury. (2010) 41:894–8. 10.1016/j.injury.2010.02.023 21574279

[8] Sahuquillo J Arikan F Decompressive craniectomy for the treatment of refractory high intracranial pressure in traumatic brain injury. Cochr Database Systemat Rev. 2006:Cd003983 10.1002/14651858.CD003983.pub2 16437469

[9] Bohman LE Schuster JM. Decompressive craniectomy for management of traumatic brain injury: an update. Curr Neurol Neurosci Rep. (2013) 13:392. 10.1007/s11910-013-0392-x 24101348

[10] Huang X Wen L. Technical considerations in decompressive craniectomy in the treatment of traumatic brain injury. Int J Med Sci. (2010) 7:385–90. 10.7150/ijms.7.385 21103073 PMC2990073

[11] Hutchinson PJ Kolias AG Timofeev IS Corteen EA Czosnyka M Timothy J. Trial of decompressive craniectomy for traumatic intracranial hypertension. N Engl J Med. (2016) 375:1119–30. 10.1056/NEJMoa1605215 27602507

[12] Cooper DJ Rosenfeld JV Murray L Arabi YM Davies AR D'Urso P. Decompressive craniectomy in diffuse traumatic brain injury. N Engl J Med. (2011) 364:1493–502. 10.1056/NEJMoa1102077 21434843

[13] Kolias AG Kirkpatrick PJ Hutchinson PJ. Decompressive craniectomy: past, present and future. Nat Rev Neurol. (2013) 9:405–15. 10.1038/nrneurol.2013.106 23752906

[14] Huang AP Tu YK Tsai YH Chen YS Hong WC Yang CC. Decompressive craniectomy as the primary surgical intervention for hemorrhagic contusion. J Neurotra. (2008) 25:1347–54. 10.1089/neu.2008.0625 19061378

[15] Soukiasian HJ Hui T Avital I Eby J Thompson R Kleisli T. Decompressive craniectomy in trauma patients with severe brain injury. Am Surg. (2002) 68:1066–71. 10.1007/s00701-017-3418-3 12516810

[16] Akyuz M Ucar T Acikbas C Kazan S Yilmaz M Tuncer R. Effect of early bilateral decompressive craniectomy on outcome for severe traumatic brain injury. Turkish Neurosurg. (2010) 20:382–9. 10.5137/1019-5149.JTN.2785-09.1 20669113

[17] Wen L Wang H Wang F Gong JB Li G Huang X. A prospective study of early versus late craniectomy after traumatic brain injury. Brain Injury. (2011) 25:1318–24. 10.3109/02699052.2011.608214 21902550

[18] Lu LQ Jiang JY Yu MK Hou LJ Chen ZG Zhang GJ. Standard large trauma craniotomy for severe traumatic brain injury. Chin J Traumatol. (2003) 6:302–4. 14514369

[19] Gillett GR Honeybul S Ho KM Lind CR. Neurotrauma the RUB: where tragedy meets ethics and science. J. Med. Ethics. (2010) 36:727–30. 10.1136/jme.2010.037424 20852302

[20] Honeybul S Ho KM. The current role of decompressive craniectomy in the management of neurological emergencies. Brain Inj. (2013) 27:979–91. 10.3109/02699052.2013.794974 23662706

[21] Kiphuth IC Köhrmann M Lichy C Schwab S Huttner HB. Hemicraniectomy for malignant middle cerebral artery infarction: retrospective consent to decompressive surgery depends on functional long-term outcome. Neurocrit Care. (2010) 13:380–4. 10.1007/s12028-010-9449-8 20890678

[22] Larach DR Larach DB Larach MG. A life worth living: seven years after craniectomy. Neurocrit. Care. (2009) 11:106–11. 10.1007/s12028-008-9180-x 19247839

[23] Ho KM Honeybul S Litton E. Delayed neurological recovery after decompressive craniectomy for severe non-penetrating traumatic brain injury. Crit Care Med. (2011) 39:2495–500. 10.1097/CCM.0b013e318225764e 21705893

[24] Gouello G Hamel O Asehnoune K Bord E Robert R Buffenoir K. Study of the long-term results of decompressive craniectomy after severe traumatic brain injury based on a series of 60 consecutive cases. Scient World J. (2014) 2014:207585. 10.1155/2014/207585 24719566 PMC3956416

[25] Molyneux AJ Kerr RS Birks J Ramzi N Yarnold J Sneade M. Risk of recurrent subarachnoid haemorrhage, death, or dependence and standardised mortality ratios after clipping or coiling of an intracranial aneurysm in the International Subarachnoid Aneurysm Trial (ISAT): long-term follow-up. Lancet Neurol. (2009) 8:427–33. 10.1016/S1474-4422(09)70080-8 19329361 PMC2669592

[26] Hoofien D Gilboa A Vakil E Donovick PJ. Traumatic brain injury (TBI) 10–20 years later: a comprehensive outcome study of psychiatric symptomatology, cognitive abilities and psychosocial functioning. Brain Injury. (2001) 15:189–209. 10.1080/026990501300005659 11260769

[27] Stocchetti N Zanier ER. Chronic impact of traumatic brain injury on outcome and quality of life: a narrative review. Criti Care. (2016) 20:148. 10.1186/s13054-016-1318-1 27323708 PMC4915181

[28] Djurisic S Rath A Gaber S Garattini S Bertele V Ngwabyt S-N. Barriers to the conduct of randomised clinical trials within all disease areas. Trials. (2017) 18:360. 10.1186/s13063-017-2099-9 28764809 PMC5539637

[29] Llewellyn-Bennett R Bowman L Bulbulia R. Post-trial follow-up methodology in large randomized controlled trials: a systematic review protocol. System Rev. (2016) 5:214. 10.1186/s13643-016-0393-3 27978859 PMC5159967

[30] Piedra MP Nemecek AN Ragel BT. Timing of cranioplasty after decompressive craniectomy for trauma. Surg Neurol Int. (2014) 5:25. 10.4103/2152-7806.127762 24778913 PMC3994696

